# Fatal Events Associated with Adverse Drug Reactions in the Korean National Pharmacovigilance Database

**DOI:** 10.3390/jpm12010005

**Published:** 2021-12-21

**Authors:** Hyeong-Geun Jo, Kyeoul Jeong, Ji-Young Ryu, Soyun Park, Yun-Seok Choi, Won-Gun Kwack, Yeo-Jin Choi, Eun-Kyoung Chung

**Affiliations:** 1 Department of Pharmacy, College of Pharmacy, Kyung Hee University, Seoul 02447, Korea; meerkatjojo@khu.ac.kr (H.-G.J.); kyeoul93@khu.ac.kr (K.J.); neo_anubis@naver.com (J.-Y.R.); 1223psy@naver.com (S.P.); cys2013@msn.com (Y.-S.C.); 2 Division of Pulmonary, Allergy and Critical Care Medicine, Kyung Hee University Hospital, Seoul 02447, Korea; wongunnim@naver.com; 3 Department of Clinical Pharmacy, Graduate School of Clinical Pharmacy, CHA University, Seongnam 13488, Korea; 4 Department of Pharmacy, Kyung Hee University Hospital at Gangdong, Seoul 05278, Korea; 5 Department of Regulatory Science, Graduate School, Kyung Hee University, Seoul 02447, Korea

**Keywords:** adverse drug reactions, death, drug safety, fatal events, KAERS, patient safety, pharmacovigilance

## Abstract

Adverse drug reactions (ADRs) pose a global public health threat, substantially contributing to death. Due to the relative paucity of clinical evidence regarding fatal ADRs, this study was performed to characterize the epidemiology of fatal ADRs in Korea. This was a retrospective, cross-sectional analysis of ADR cases reported to the Korea Adverse Event Reporting System from 2010 to 2019. All ADRs were coded using the World Health Organization-Adverse Reaction Terminology system and classified as either fatal or non-fatal events. Logistic regression was performed to identify factors associated with fatal events. Among 289,756 ADR records, 629 fatal events (0.2%) occurred. The most common causative agent of fatal ADRs was antibacterials (20.3%), followed by antimycobacterials (5.4%), analgesics (4.0%), and contrast media (1.9%). Among antimicrobials, vancomycin was most frequently implicated without significantly increasing the risk of fatal events. The risk for fatal ADRs was significantly increased with male sex; advanced age; polypharmacy; piperacillin/β-lactamase inhibitor; cefotetan; ceftriaxone; combination antimycobacterial therapy consisting of rifampicin, isoniazid, pyrazinamide, and ethambutol; morphine; and iopromide (reporting odds ratio > 1, *p* < 0.05 for all). Although fatal ADRs are uncommon (<1%) in Korea, they are primarily caused by commonly used medications including antibiotics, analgesics, and contrast media.

## 1. Introduction

Adverse drug reactions (ADRs) pose a substantial global burden in healthcare and medicine, endangering patient safety. According to the Global Burden of Disease Study, ADRs commonly occur with the reported prevalence and incidence of 2,673,100 and 34,975,000 in 2017, respectively [1]. The majority of ADRs are temporary and minor; however, a growing proportion of patients may experience sentinel events associated with drug therapy, potentially resulting in hospitalizations or even death [2]. According to the Food and Drug Administration (FDA), the number of serious adverse event reports has been consistently increasing every year, leading to a 2.6-fold increase in the total serious adverse event reports from 2006 to 2014 [3]. Previous studies suggested adverse drug events (ADEs) as one of the most common causes of hospitalization, accounting for approximately 5% to 10% of total hospital admissions [4,5]. The number of fatal ADE cases reported to the FDA has been continuously increasing with a 2.7-fold increase over the period of 2006 to 2014 [3], accounting for the fourth to sixth leading cause of death [6].

As the population ages, the prevalence of chronic diseases is increasing with more medication therapy required. This contributes to the increasing prevalence of polypharmacy associated with a high risk of serious ADRs including fatal events. Thus, for safe and effective medication use, the epidemiologic characteristics of serious ADEs, particularly drug-related fatal events, should be elaborated including causative agents, associated ADRs, and risk factors. Currently, only a few previously published studies evaluated fatal ADEs [6,7,8,9,10,11,12,13]. However, approximately half of the studies were conducted before 2010, highlighting the urgent need for updated information regarding fatal ADRs. A recently published study assessed the association between fatal ADEs and various potentially etiologic medications, including anti-cancer medications, suggesting oncologic drugs as one of the most common causative medications of fatal ADRs [6]. However, the inclusion of oncologic medications as potential offending agents leaves the interpretation of the study results challenging due to substantial mortality associated with cancer. Furthermore, fatal ADE cases have never been analyzed in Korea except for opioid drug abuse or overdose cases [6,14]. Considering the variable demographic, clinical, and sociocultural aspects associated with medication use in clinical practice, timely assessment of the real-world data for medication use in a specific country and/or ethnic group may assist in preventing excessive fatal ADEs and ultimately, promoting patient safety.

In addition to fatal ADEs, non-fatal ADEs might adversely affect patient outcomes including increased morbidity and decreased quality of life. A recent meta-analysis suggested ADEs as a major cause of hospitalizations leading to complicated hospital course [12]. Moreover, according to a long-term, prospective safety study, approximately 20% of serious ADEs resulting in hospitalization were preventable, primarily associated with using potentially inappropriate medications in the elderly population [15]. In an effort to ensure patient safety, voluntary ADR reporting systems have been constructed in many countries; in Korea, the first spontaneous ADR report was received in 1988 [16]. The voluntary ADR reporting system is one of the real-world data (RWD) sources commonly used for post-approval pharmacovigilance assessment based on the real-world pattern of medication utilization. However, under-reporting is the major drawback of using the spontaneous ADR reports for reliable and accurate data analysis due to lack of interest or time, ignorance on non-serious ADRs, and limited pharmacovigilance knowledge [17,18]. In fact, the annual number of ADR reports was <100,000 cases per year in Korea until 2012, suggesting substantial under-reporting of ADRs. With the Korea Adverse Event Reporting System (KAERS) constructed by the Korea Institute of Drug Safety and Risk Management (KIDS) in 2012 as a voluntary ADR reporting system, spontaneous ADR reports have been systematically collected and evaluated in collaboration with regional pharmacovigilance centers (RPVCs) [16,19,20]. The annual number of ADR reports was exponentially increased in 2013 to 183,260 when at least 22 RPVCs were designated [20], highlighting significantly improved data quality to assess medication safety based on voluntary ADR reporting data in the KAERS throughout the pharmaceutical product life cycle. 

Despite rigorous investigation of medication safety in pre-approval clinical trials, only limited evidence regarding safety of the medication might be available from these studies due to limited study population and relatively short-term use of the medication. In order to assess the real world, long-term safety of medications used in clinical practice, analysis of RWD is pertinent. Although the National Health Insurance service sample (NHISS) cohort data is the most frequently used RWD source in Korea, due to the national health insurance system, it does not contain non-reimbursement data records. Thus, voluntary ADE reports may be a more appropriate and comprehensive RWD source to evaluate fatal ADRs because (1) they include ADEs associated with non-reimbursed as well as reimbursed medications, and (2) healthcare practitioners are legally required to report clinically serious ADEs, such as death. Therefore, the aims of this study were to characterize the prevalence and causes of fatal ADEs in Korea and to identify risk factors associated with fatal ADEs based on the national pharmacovigilance database in Korea over the period of 10 years.

## 2. Results

### 2.1. Baseline Demographics

A total of 289,756 ADE records reported to the KAERS from January 2010 to December 2019 were included in this study. Among them, drug-related fatal events accounted for 629 (0.2%) reports. The baseline demographic characteristics of patients included in the analysis are summarized in Table 1. While non-fatal ADRs were more likely to occur in females [*n* = 164,621 (56.9%)], fatal events were predominantly reported in males [*n* = 361 (57.4%)]. Both fatal and non-fatal ADRs were most frequently reported in patients at the age of 70 years or older [*n* = 214 (34.0%) for fatal and *n* = 69,965 (24.2%) for non-fatal cases]. The majority of the non-fatal and fatal ADRs were reported by nurses (*n* = 127,962; 44.3%) and physicians (*n* = 266; 42.3%), respectively.

### 2.2. Etiologic Medications Implicated in Fatal Events 

The causative agents and drug classes significantly associated with death are summarized in Table 2; Supplementary Table S1 shows the comprehensive results of the estimated risk of fatal events associated with all included medications in our present analysis. Drug-related fatal events were reported at the highest relative frequency for antibacterial drugs (20.3%; reporting odds ratio [ROR] 1.432, 95% confidence interval [CI] 1.179–1.740), followed by antimycobacterial agents (5.4%; ROR 2.390, 95% CI 1.691–3.377), analgesics (3.9%; ROR 2.484, 95% CI 1.665–3.706), and contrast media (1.9%; ROR 4.274, 95% CI 2.358–7.770). Among antibacterial agents, piperacillin/β-lactamase inhibitor (e.g., tazobactam) combination was most frequently associated with antibacterial-related deaths (*n* = 18; ROR 1.685, 95% CI 1.054–2.694), followed by ceftriaxone (*n* = 9; ROR 4.617, 95% CI 2.391–8.917) and cefotetan (*n* = 3; ROR 3.859, 95% CI 1.241–12.002). Most of antimycobacterial- and contrast media-induced fatal events were reported with the first-line antimycobacterial agents (*n* = 33 out of 34), including rifampicin (ROR 2.079, 95% CI 1.113–3.884), isoniazid (ROR 1.991, 95% CI 1.066–3.721), ethambutol (ROR 2.743, 95% CI 1.302–5.779), pyrazinamide (ROR 2.622, 95% CI 1.173–5.861), and iopromide (*n* = 11 out of 12; ROR 4.280, 95% CI 2.358–7.770), respectively. Morphine was the most common culprit agent associated with analgesic-related fatal cases (*n* = 7 out of 25; ROR 4.779, 95% CI 2.269–10.068). 

**Figure 1:**
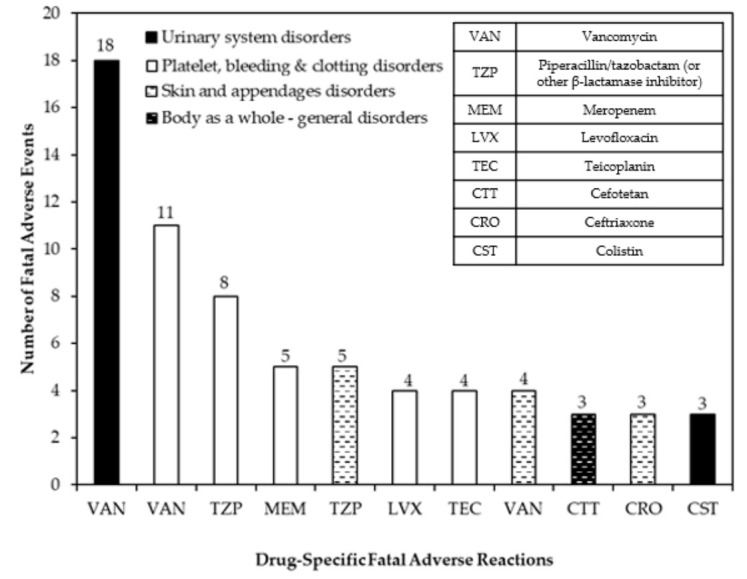
Specific antibiotic-induced adverse reactions resulting in at least three fatal cases over the study period.

Among the fatal adverse drug reactions associated with antibacterial agents, specific antibiotic-induced adverse reactions leading to at least three fatal cases over the study period are summarized in Figure 1. All of the reported fatal adverse reactions associated with the antibacterial agents are typical of each medication as stated in their prescribing information [21]. The most predominant drug-specific fatal adverse reaction was vancomycin-induced urinary system disorders (e.g., nephrotoxicity) (*n* = 18); followed by platelet, bleeding, and clotting disorders (e.g., thrombocytopenia) caused by vancomycin (*n* = 11) and piperacillin/β-lactamase inhibitor (*n* = 8). Although much less common, the following immune-mediated, hypersensitivity reactions were also implicated in death: skin and appendages disorders (e.g., skin eruption) induced by piperacillin/β-lactamase inhibitor (*n* = 5), vancomycin (*n* = 4), and ceftriaxone (*n* = 3); and general disorders (e.g., anaphylactic shock) resulting from cefotetan (*n* = 3).

### 2.3. Factors Associated with Drug-Induced Fatal Events 

The univariate analysis showed significant association of drug-induced fatal events with sex, age, the number of concomitantly used medications, and the offending agent (*p* < 0.05, Table 3). All of these factors remained significantly associated with the risk of drug-related death in the multivariate regression analysis (*p* < 0.05, Table 3). Fatal adverse reactions were more likely to be reported in the following: males (ROR 1.894, 95% CI 1.616–2.222, *p* < 0.001); individuals with advanced age (ROR 1.013, 95% CI 1.009–1.018, *p* < 0.001); patients taking multiple medications (ROR 1.072, 95% CI 1.052–1.092, *p* < 0.001); and those treated with piperacillin/β-lactamase inhibitor (ROR 2.255, 95% CI 1.404–3.621, *p* = 0.001), cefotetan (ROR 3.991, 95% CI 1.280–12.440, *p* = 0.017), ceftriaxone (ROR 5.218, 95% CI 2.694–10.107, *p* < 0.001), morphine (ROR 4.783, 95% CI 2.264–10.103, *p* < 0.001), iopromide (ROR 4.649, 95% CI 2.550–8.473, *p* < 0.001), and the four-drug combination antimycobacterial regimen consisting of rifampicin, isoniazid, pyrazinamide, and ethambutol (RIPE) (ROR 3.238, 95% CI 2.276–4.605, *p* < 0.001). Each antimycobacterial component of the RIPE regimen was significantly associated with the risk of fatal adverse drug reactions in the univariate analysis; however, only the RIPE regimen, but not each individual antimycobacterial agent, significantly increased the risk of drug-induced fatal events in the multivariate analysis (Table 3).

## 3. Discussion

Adverse drug events pose a substantial threat to public health and patient safety [6,22]. The majority of ADEs are mild to moderate, resulting in no harm to reversible harm to patients. The proportion of serious ADEs including life-threatening events and death is not negligible, reported from < 1% up to 67.0% depending on the study population and medications [23,24,25,26,27]. Despite the significance of serious ADEs, few published pharmacovigilance data are available focusing on drug-induced fatal cases [3,6,9,10]. Our current study characterized fatal adverse events associated with medications based on spontaneous ADE reporting data in Korea from 2010 to 2019. Similar to previous studies [6,9], the incidence of drug-related fatal events was not negligible [0.2% (*n* = 629) of all ADE reports (*n* = 289,127)]; however, our estimated incidence was much lower than that in previous studies (1.34–3.88%) [3,6,9]. The lower rate of drug-induced death in our present study might be attributed to excluding ADE cases caused by antineoplastic agents (WHO Anatomical Therapeutic Chemical (ATC) classification system code of L01) in an attempt to minimize confounding effects associated with cancer. In fact, a recent study investigating fatal ADRs based on the WHO pharmacovigilance database suggested anticancer drugs as the most common causative agent of fatal ADRs [6]. Furthermore, our study only included ADR cases with causality assessed as certain, probable/likely, or possible to ensure significant contribution of the reported etiologic medication to the fatal event. Overall, the exclusion of ADE reports associated with oncologic medications and those with causality graded lower than possible might lead to a lower rate of drug-related fatal events compared to previous studies [3,6,9,10]. However, their exclusion minimized confounding effects associated with underlying oncologic diseases as well as other potential causes of death than a drug, which might suggest our estimated incidence of drug-induced death to be more robust and reliable. Future large-scale studies, preferably prospective studies, are warranted to determine a more precise and accurate incidence of drug-related fatal events.

Similar to previous studies, our present study suggested an increased risk of drug-related fatal events in males, elderly patients, and patients concurrently using multiple medications, which was confirmed in the univariate and subsequent multivariate analysis [28,29,30]. While aging and polypharmacy have been extensively reported in previous studies as major risk factors of serious ADRs [28,29], the increased risk of fatal ADRs in males compared to females could not be clearly explained from the physiological standpoint. However, this difference in the risk of drug-induced fatal events between genders might be accounted for by the different attitudes toward health [31,32]. Actually, according to a previous study, men tended to perceive their health as good as women, despite overall worse health status when measured objectively. In addition to sex, age, and the number of concurrently used medications, individuals with more comorbidities might be at an increased risk of fatal ADRs. However, due to extensive missing information regarding comorbid diseases in the KAERS data (44.0% to 58.2%), the association of the fatal ADR risk and the number and/or severity of underlying diseases could not be examined in the multivariate analysis. Additional studies with more comprehensive, large-scale data are imperative to investigate the impact of the number and/or severity of underlying diseases on the risk of drug-related fatal events.

In our current study, antibacterial drugs were most frequently associated with drug-induced fatal events. This is consistent with a previous pharmacovigilance study conducted in Italy, suggesting systemic anti-infectives as the most common medications involved in the fatal ADRs [9]. In contrast to the Italian study, our study showed significantly increased risk for fatal events with antibacterial agents (ROR (95% CI); 1.432 (1.179–1.740) vs. 0.48 (0.39–0.58)). One of the possible reasons for the significantly increased risk of antibacterial-induced fatal events was relatively high rates of antibiotic hypersensitivity reactions such as skin/appendages disorders and anaphylactic general disorders, primarily associated with beta-lactams (e.g., piperacillin/β-lactamase inhibitor, ceftriaxone, and cefotetan). This is consistent with previous studies suggesting significantly increased risk of drug hypersensitivity reactions, including beta-lactam allergy, in Asian-Pacific ethnic groups, including Koreans compared to Caucasians [33,34]. Additionally, relatively high incidence of antibiotic-associated thrombocytopenia in our study might contribute to the significantly increased risk of antibacterial-related fatal events. Considering the dose-dependent risk of antibiotic-induced thrombocytopenia, fatal platelet disorders might result from prevalent dosing errors of antibiotics as suggested in a previous study [35]. This might account for the highest frequency of vancomycin-induced nephrotoxicity among drug-specific fatal reactions; however, the risk of fatal events was not significantly increased with vancomycin. This suggests non-fatal adverse events including nephrotoxicity occur much more frequently than fatal cases, as documented in the vancomycin prescribing information as well as previous studies [21,36]. Overall, the significantly higher incidence of antibiotic-induced fatal events in this study might be associated with an increased risk for drug hypersensitivity reactions in the Asian-Pacific ethnic group including Koreans and the high prevalence of antibiotic dosing errors leading to fatal platelet disorders. Further large-scale comparative studies are required to confirm the association of fatal events caused by antibiotics with ethnic risk of drug hypersensitivity reactions as well as inappropriate antibiotic dosing.

To our knowledge, this is the first study showing a significantly increased risk of fatal adverse reactions associated with antimycobacterial agents. The relatively high prevalence and significantly increased risk of fatal ADEs caused by antimycobacterial agents might be associated with the high prevalence and incidence of tuberculosis in Korea [37,38,39]. In 2018, the reported average incidence of tuberculosis among countries of the Organisation for Economic Co-operation and Development (OECD) was 9 cases per 100,000 population, which was much lower than the incidence in Korea (62 cases per 100,000) [40]. The high disease burden of tuberculosis in Korea results in higher prevalence of anti-tuberculosis medication use, which might contribute to the reported high risk of fatal ADRs induced by anti-tuberculosis medications [39]. Moreover, as suggested in a previous study, the relatively lower body mass among Koreans might lead to substantially larger systemic exposures of antimycobacterial agents, consequently increasing the risk of AEs including fatal ADRs [41]. Our multivariate analysis showed a significantly increased risk of fatal ADRs with the RIPE regimen, but not with each antimycobacterial agent in the RIPE regimen. This is consistent with previous study findings because hepatotoxicity commonly occurs with anti-tuberculosis medications such as isoniazid, rifampicin, and pyrazinamide; thus, use of more than one hepatotoxic antimycobacterial agent increases the incidence as well as the severity of anti-tuberculosis ADRs, potentially leading to fatal events [42]. In contrast to a previous study suggesting long-term INH treatment as a risk factor for serious INH-induced hepatotoxicity, INH, which is commonly used for at least 6 months, was not significantly associated with the risk of fatal ADRs in our multivariate analysis when used alone. This may be associated with a relatively low frequency of *NAT2* slow acetylator alleles in Koreans compared to other ethnic groups, specifically Caucasians (32% vs. 76%) [43]; the slower clearance of INH in slow acetylators results in the accumulation of INH, consequently increasing the risk of INH-induced hepatotoxicity including fatal AEs [44]. Overall, patients receiving the four-drug RIPE regimen, especially over a prolonged period of time, should be monitored closely in clinical practice to prevent potentially fatal ADRs. Future large-scale studies may assist in identifying individuals at high risk of antimycobacterial-related death by accounting for various genetic, demographic, and clinical characteristics.

Consistent with previous studies [23,25,28,45], analgesics were one of the most common medication classes implicated in fatal ADRs with morphine as the only analgesic agent significantly increasing the risk of fatal adverse events. This finding is similar to our previous study results suggesting opioids including morphine account for the majority of analgesic-related deaths [28]. The prevalent opioid-related deaths might be closely associated with the current opioid epidemic characterized by opioid abuse and overuse [46,47]. As previous studies suggested, opioid-induced oversedation frequently results in serious adverse events including respiratory depression and cardiovascular toxicities such as hypotension, potentially leading to death [48,49]. However, caution should be exercised when interpreting the increased risk of morphine-related death reported in our current study. Considering morphine is one of the most commonly prescribed analgesic medications in terminally ill patients, the significantly increased risk of fatal events associated with morphine might be confounded by the underlying diseases of morphine users. In fact, potentially fatal ADRs of opioids such as respiratory dysfunction and cardiovascular collapse are common clinical manifestations of terminal illness [50]. Unfortunately, due to extensive missing information regarding comorbidities in the KAERS database, the impact of the patient’s underlying diseases such as terminal cancer could not be adjusted when estimating the risk of morphine-induced fatal ADRs in our present study. Overall, morphine should be used judiciously by evaluating the therapeutic benefit versus risk based on patient characteristics such as comorbid diseases.

Our present study suggested contrast media implicated in substantial fatal ADR reports. The majority of contrast media-induced fatal cases resulted from iopromide, which is a nonionic, low-osmolar, iodinated contrast media agent commonly used for X-ray imaging studies in clinical practice [51]. Similarly, a previous pharmacovigilance study in Korea reported iopromide as the most common causative agent of ADRs among seven iodinated contrast media [51]. This finding should be interpreted with caution because iopromide accounts for the largest share of the contrast media market in practice [51], potentially leading to the highest rate of iopromide-induced ADR reports. Although the majority of the contrast media-induced AEs are mild and reversible including nausea, vomiting, itching, and nephrotoxicity, ADRs caused by radiocontrast media might result in life-threatening or even fatal events such as hypotensive shock, respiratory arrest, cardiac arrest, and convulsions [51]. The increased risk for fatal ADRs caused by contrast media might be associated with the high risk of drug hypersensitivity reactions including radiocontrast media hypersensitivity in Asian-Pacific ethnic groups such as Koreans compared to Caucasians [33,34,52]. In fact, a previous study comparing the safety profiles of seven iodinated contrast media in Korea showed iopromide-induced skin reactions such as urticaria as the most frequently reported ADE [51], which is a common clinical manifestation of radiocontrast media hypersensitivity reaction [52,53]. Considering the high risk of developing severe and even fatal ADRs associated with contrast media including radiocontrast hypersensitivity reactions, close monitoring should be implemented for patients receiving at least one dose of contrast media agents for prompt and appropriate management of potentially fatal ADRs due to contrast media. 

Some limitations should be considered when evaluating the results of this study. First, because the KAERS database is a spontaneous reporting system, under-reporting of AEs could occur although it is unlikely for fatal cases due to the required case reporting for compensatory relief of injury from ADRs [54,55]. Nevertheless, the total number of fatal adverse event cases was relatively small; thus, statistical analysis was performed for limited medications and drug classes to ensure adequate robustness and reliability. In addition, the number of safety reports for a specific etiologic agent might be substantially affected by the amount of medication use in clinical practice, highlighting the need to carefully interpret our current study findings. Lastly, considering the nature of the study design as well as extensive missing information to characterize the reported ADRs such as indications, comorbid diseases, and concurrent medications, our present study should not be used to determine a definite causality between the reported fatal event and the offending medication. In order to minimize the effects of potential confounding factors such as underlying diseases on the risk of fatal ADRs, ADE reports associated with oncologic medications were excluded in our study. Considering antineoplastic agents reported as the most frequently implicated class in fatal ADRs according to a recent global pharmacovigilance study [6], additional studies with more comprehensive real-world data including anticancer drug safety data are imperative for more accurate and precise characterization of fatal ADRs. Although the quality of recorded information in the safety reports was considered adequate through data verification by a reporter and an independent record reviewer in the KIDS, future large-scale, preferably prospective, studies are warranted to elaborate the impact of various nondrug factors or other medications than the reported culprit drug on the risk of fatal ADRs. 

## 4. Materials and Methods

### 4.1. Data Source and Definition

In this retrospective pharmacovigilance study, ADR reports voluntarily submitted to the KAERS over the period of January 2010 to December 2019 were evaluated to compare epidemiologic characteristics of fatal and non-fatal ADRs. The KAERS is the Korean national pharmacovigilance database consisting of spontaneous ADR reports forwarded to the KIDS. All individual ADRs were recorded using standardized case reporting forms, developed and validated by the KIDS, to document the following information: (1) patient demographics including age, sex, and comorbidities; (2) medication information including concomitant medications, dates of treatment initiation and cessation, and the offending agent; (3) ADR information including the year of case reports and causality assessment between the documented ADR and the causative drug; and (4) seriousness of adverse events [19]. Each ADR report was submitted by the general public, healthcare professionals, and pharmaceutical companies [16,19]. The submitted ADR reports were further reviewed and verified by healthcare professionals in the KIDS prior to deposition into the KAERS.

The included ADR reports in this study were individual case safety reports submitted to the KAERS from January 2010 to December 2019 and those with causality between the ADR and etiologic drugs assessed as “certain”, “probable/likely”, or “possible” according to the World Health Organization-Uppsala Monitoring Centre (WHO-UMC) criteria [56]. The ADR reports were grouped into either fatal events or non-fatal events. Fatal events were defined as the individual ADR case safety reports documented to result in death based on the International Conference on Harmonization (ICH) E2D Guideline [57]. Non-fatal events were the ADR reports that did not meet the criteria for fatal events. Individual case safety reports associated with drugs exclusively leading to non-fatal events were excluded from this comparative analysis of fatal vs. non-fatal ADRs. Causative medications associated with reported ADRs were classified according to the WHO ATC classification system up to the second level. All ADRs were recorded based on the WHO-Adverse Reaction Terminology (WHO-ART) as preferred terms or included terms [58], which were grouped into System Organ Class (SOC) disorders. In an effort to minimize the obscure relationship between offending agents and adverse events, particularly fatal outcomes, the ADR cases reportedly caused by antineoplastic agents with the WHO ATC code of L01 were excluded due to the substantial disease severity of underlying malignant tumors [59].

The study protocol for using the KAERS database was approved by the KIDS (No. 2006A0035, No. 2007A0058) and the institutional review board of Kyung Hee University (No. KHSIRB-20-314) (Seoul, Korea). Informed consents were exempted by the board.

### 4.2. Statistical Analysis 

For statistical analyses, SPSS Statistics 25.0 (IBM SPSS Statistics for Windows, Armonk, NY) were used. Descriptive statistics were utilized to summarize baseline characteristics of fatal and non-fatal cases of ADR reports with mean ± standard deviation. Categorical variables were presented as absolute and relative frequencies. Comparison between fatal and non-fatal reports was performed utilizing the Mann–Whitney *U* test for non-normally distributed continuous data, unpaired 2-tailed *t* test for normally distributed continuous data, and the χ^2^ or the Fisher’s exact test for categorical data. Normality for continuous variables was tested using the Kolmogorov-Smirnov test.

The association between death and causative drugs or drug classes was assessed using disproportionality analysis based on the ADR reporting odds ratios (RORs) with the corresponding 95% confidence intervals (CIs). For reliable analyses, RORs were estimated for specific offending medications or drug classes with at least three reported fatal cases [28,60,61,62]. Disproportionality was defined as the lower limit of the ROR 95% CI greater than 1, which was considered to robustly increase the risk for fatal ADRs [60,63]. 

In order to identify factors significantly associated with risk for drug-related fatal events, the univariate analysis was first performed for the following factors: sex, age, implicated drug, and the number of concurrently used medications. Multiple logistic regression analysis was then conducted using a stepwise forward method by examining the factors significantly associated with fatal cases in the univariate analysis. Variables with *p* < 0.1 in the univariate analysis and clinical plausibility were further tested in the multiple logistic regression. Statistical significance was defined as *p* value < 0.05 throughout statistical analyses.

## 5. Conclusions

The prevalence of fatal events associated with medications is relatively low in Korea (0.2%). The risk of fatal ADRs is significantly increased with antibacterial drugs, antimycobacterials, analgesics, and contrast media. Antibacterial agents are the most common cause of fatal ADRs, among which vancomycin is most frequently involved in fatal adverse events without significantly increasing the risk of fatal events vs. non-fatal ADRs. Fatal adverse drug events are more likely to occur in male, elderly patients receiving multiple medications, especially when treated with piperacillin/β-lactamase inhibitor; cefotetan; ceftriaxone; four-drug anti-tuberculosis regimen consisting of rifampicin, isoniazid, pyrazinamide, and ethambutol; morphine; and iopromide (an iodinated contrast media agent). Large, national pharmacovigilance database is a unique tool to elaborate the epidemiologic characteristics of fatal ADRs in the country or ethnic group considering specific demographic, clinical, and sociocultural characteristics of medication use in clinical practice. Further studies evaluating fatal ADRs based on more comprehensive pharmacovigilance data analysis are warranted to promote safe medication use in clinical practice, ultimately ensuring patient safety.

## Figures and Tables

**Table 1:** Baseline demographic characteristics of patients included in this study (*n* = 289,756) ^a^.

Characteristics	Fatal Case (*n* = 629)	Non-Fatal Case (*n* = 289,127)
Sex		
Male	361 (57.4)	124,506 (43.1)
Female	268 (42.6)	164,621 (56.9)
Age (Years)		
<10	16 (2.5)	7758 (2.7)
10–19	9 (1.4)	8319 (2.9)
20–29	13 (2.1)	17,667 (6.1)
30–39	23 (3.7)	28,589 (9.9)
40–49	96 (15.3)	39,211 (13.6)
50–59	129 (20.5)	59,357 (20.5)
60–69	129 (20.5)	58,261 (20.2)
≥70	214 (34.0)	69,965 (24.2)
Causality ^b^		
Certain	1 (0.2)	7025 (2.4)
Probable/likely	102 (16.2)	80,917 (28.0)
Possible	526 (83.6)	201,185 (69.6)
Individuals reporting adverse events ^c^		
Physicians	266 (42.3)	47,138 (16.3)
Pharmacists	51 (8.1)	76,171 (26.3)
Nurses	47 (7.5)	127,962 (44.3)
General Public	32 (5.1)	6787 (2.3)
Others	135 (21.5)	15,490 (5.4)
Number of concurrently used medications		
1	197 (31.3)	122,920 (42.5)
2	95 (15.1)	39,434 (13.6)
3	120 (19.1)	36,842 (12.7)
4	52 (8.3)	35,579 (12.3)
≥5	165 (26.2)	54,352 (18.8)
Number of comorbidities ^d^		
1	152 (24.2)	150,842 (52.2)
2	44 (7.0)	7044 (2.4)
3	29 (4.6)	2396 (0.8)
4	5 (0.8)	924 (0.3)
≥5	33 (5.2)	847 (0.3)

^a^ Data presented as the number of cases (% relative frequency). ^b^ Causality was assessed according to the World Health Organization—Uppsala Monitoring Centre (WHO-UMC) criteria. ^c^ Information was missing in 98 (15.6%) fatal and 15,579 (5.4%) non-fatal cases, respectively. ^d^ Information was missing in 366 (58.2%) fatal and 127,074 (44.0%) non-fatal cases, respectively.

**Table 2:** Causative agents significantly associated with fatal adverse drug reactions.

WHO-ATC Code ^a^	Therapeutic Class or Agent	Number (%) of Reported Fatal Events (*n* = 629)		ROR (95% CI) ^b^	*p*-Value ^c^
		Number of Fatal Events	Relative Frequency		
J01	Antibacterial drugs	128	20.3%	1.432 (1.179–1.740)	<0.001
	Piperacillin/BLI	18	2.9%	1.685 (1.054–2.694)	0.029
	Ceftriaxone	9	1.4%	4.617 (2.391–8.917)	<0.001
	Cefotetan	3	0.5%	3.859 (1.241–12.002)	0.020
J04	Antimycobacterials	34	5.4%	2.390 (1.691–3.377)	<0.001
	Rifampicin	10	1.6%	2.079 (1.113–3.884)	0.022
	Isoniazid	10	1.6%	1.991 (1.066–3.721)	0.031
	Ethambutol	7	1.1%	2.743 (1.302–5.779)	0.008
	Pyrazinamide	6	1.0%	2.622 (1.173–5.861)	0.019
N02	Analgesic drugs	25	4.0%	2.484 (1.665–3.706)	<0.001
	Morphine	7	1.1%	4.779 (2.269–10.068)	<0.001
V08	Contrast media	12	1.9%	4.274 (2.413–7.568)	<0.001
	Iopromide	11	1.7%	4.280 (2.358–7.770)	<0.001

Abbreviations: WHO-ATC, World Health Organization-Anatomical Therapeutic Chemical Classification System; ROR, reporting odds ratio; CI, confidence interval; BLI, beta-lactamase inhibitor. ^a^ WHO-ATC code shown up to the second level (bolded). ^b^ ROR from the Mantel–Haenszel test for fatal events compared to non-fatal events. ^c^ *p*-value from the Mantel–Haenszel test between fatal and non-fatal events.

**Table 3:** Univariate and multivariate analyses for the association of fatal adverse drug reactions with patient characteristics and causative medications.

	Univariate Analysis		Multivariate Analysis	
	ROR (95% CI)	*p* Value	ROR (95% CI)	*p* Value
Baseline characteristics				
Male sex	1.783 (1.520–2.088)	<0.001	1.894 (1.616–2.222)	<0.001
Age	1.015 (1.011–1.020)	<0.001	1.013 (1.009–1.018)	<0.001
Number of concurrently used medications	1.099 (1.082–1.116)	<0.001	1.072 (1.052–1.092)	<0.001
Causative medication				
Piperacillin/β-lactamase inhibitor	1.685 (1.054–2.694)	0.029	2.255 (1.404–3.621)	0.001
Cefotetan	3.859 (1.241–12.002)	0.020	3.991 (1.280–12.440)	0.017
Ceftriaxone	4.617 (2.391–8.917)	<0.001	5.218 (2.694–10.107)	<0.001
Rifampicin	2.079 (1.113–3.884)	0.022		
Isoniazid	1.991 (1.066–3.721)	0.031		
Pyrazinamide	2.622 (1.173–5.861)	0.019		
Ethambutol	2.743 (1.302–5.779)	0.008		
RIPE			3.238 (2.276–4.605)	<0.001
Morphine	4.779 (2.269–10.068)	<0.001	4.783 (2.264–10.103)	<0.001
Iopromide	4.280 (2.358–7.770)	<0.001	4.649 (2.550–8.473)	<0.001

Abbreviations: ROR, reporting odds ratio; CI, confidence interval; RIPE, four-drug combination antimycobacterial regimen consisting of rifampicin, isoniazid, pyrazinamide, and ethambutol.

## Data Availability

The datasets supporting the reported study results are available from the Korea Institute of Drug Safety and Risk Management (KIDS) for approved study protocols. Restrictions apply to the availability of these data due to the need for approval from the KIDS and the inclusion of private medical information. Data are available from the authors with permission from the KIDS.

## Supplementary Materials

The following is available online at https://www.mdpi.com/article/10.3390/jpm12010005/s1, Table S1: Medications implicated in fatal adverse drug reactions reported to the Korea Adverse Event Reporting System.
