# Supplementary material for: Fatal Events Associated with Adverse Drug Reactions in the Korean National Pharmacovigilance Database
Source: J Pers Med. 2021 Dec 21;12(1):5. doi: 10.3390/jpm12010005 (PMC8779892; doi:10.3390/jpm12010005)
Supplement: Supplementary file 1 [file jpm-12-00005-s001.zip › jpm-1483449-supplementary.pdf]

Supplementary Table S1. Medications implicated in fatal adverse drug reactions reported to the Korea Adverse Event Reporting System.

WHO-ATC Code[a]	Therapeutic Class or Agent	Number (%) of Reported Fatal Events (n = 629)		ROR (95% CI)[b]	*p*-value[c]
		Number of Fatal Events	Relative Frequency		
**J01**	**Antibacterial drugs**	**128**	**20.3%**	**1.432 (1.179–1.740)**	**< 0.001**
	Vancomycin	36	5.7%	0.712 (0.508–0.997)	0.048
	Piperacillin/BLI (combination)	18	2.9%	1.685 (1.054–2.694)	0.029
	Ceftriaxone	9	1.4%	4.617 (2.391–8.917)	< 0.001
	Meropenem	9	1.4%	1.008 (0.521–1.947)	> 0.05
	Cefotaxime	7	1.1%	1.867 (0.886–3.934)	> 0.05
	Moxifloxacin	7	1.1%	1.217 (0.577–2.566)	> 0.05
	Teicoplanin	6	1.0%	0.227 (0.101–0.510)	< 0.001
	Levofloxacin	5	0.8%	0.759 (0.314–1.831)	> 0.05
	Colistin	4	0.6%	0.146 (0.054–0.393)	< 0.001
	Linezolid	4	0.6%	0.055 (0.020–0.150)	< 0.001
	Cefotetan	3	0.5%	3.859 (1.241–12.002)	0.020
	Cefepime	3	0.5%	0.444 (0.142–1.384)	> 0.05
	Clindamycin	3	0.5%	0.319 (0.102–0.995)	0.049
**L04**	**Immunosuppressants drugs**	**80**	**12.7%**	**0.139 (0.110–0.176)**	**< 0.001**
**B01**	**Antithrombotic agents**	**50**	**7.9%**	**0.419 (0.314–0.560)**	**< 0.001**
	Cilostazol	23	3.7%	0.177 (0.116–0.268)	< 0.001
	Acetylsalicylic acid	11	1.8%	0.982 (0.541–1.784)	> 0.05
	Clopidogrel	7	1.1%	0.723 (0.343–1.526)	> 0.05
	Iloprost	5	0.8%	0.035 (0.014–0.088)	< 0.001
	Defibrotide	3	0.5%	0.009 (0.002–0.031)	< 0.001
**N05**	**Psycholeptics drugs**	**42**	**6.7%**	**0.730 (0.533–0.998)**	**0.049**
**H02**	**Corticosteroids systemic**	**41**	**6.5%**	**0.628 (0.458–0.863)**	**0.004**
**B05**	**Blood substitutes and perfusion solutions**	**36**	**5.7%**	**0.186 (0.132–0.261)**	**< 0.001**
**J04**	**Antimycobacterials**	**34**	**5.4%**	**2.390 (1.691–3.377)**	**< 0.001**
	Rifampicin	10	1.6%	2.079 (1.113–3.884)	0.022
	Isoniazid	10	1.6%	1.991 (1.066–3.721)	0.031
	Ethambutol	7	1.1%	2.743 (1.302–5.779)	0.008
	Pyrazinamide	6	1.0%	2.622 (1.173–5.861)	0.019
**N02**	**Analgesic drugs**	**25**	**4.0%**	**2.484 (1.665–3.706)**	**< 0.001**
	Acetylsalicylic acid	15	2.4%	0.790 (0.473–1.319)	> 0.05
	Morphine	7	1.1%	4.779 (2.269–10.068)	< 0.001
**M04**	**Antigout preparations**	**17**	**2.7%**	**0.206 (0.127–0.334)**	**< 0.001**
	Allopurinol	17	2.7%	0.183 (0.113–0.298)	< 0.001
**H05**	**Calcium homeostasis**	**16**	**2.5%**	**0.472 (0.287–0.776)**	**0.003**
**M01**	**Anti-inflammatory and antirheumatic drugs**	**15**	**2.4%**	**1.532 (0.918–2.558)**	**> 0.05**
	Diclofenac	10	1.6%	1.093 (0.585–2.043)	> 0.05
**N06**	**Psychoanaleptics**	**13**	**2.1%**	**0.672 (0.388–1.166)**	**> 0.05**
**V08**	**Contrast media**	**12**	**1.9%**	**4.274 (2.413–7.568)**	**< 0.001**
**J02**	**Antimycotic drugs**	**11**	**1.7%**	**0.190 (0.104–0.345)**	**< 0.001**
**L02**	**Endocrine therapy**	**11**	**1.7%**	**0.150 (0.083–0.274)**	**< 0.001**
**N03**	**Antiepileptic drugs**	**11**	**1.7%**	**0.849 (0.467–1.542)**	**> 0.05**
**V03**	**All other therapeutic products**	**10**	**1.6%**	**0.326 (0.174–0.611)**	**< 0.001**
**C08**	**Calcium channel blockers**	**8**	**1.3%**	**1.035 (0.515–2.081)**	**> 0.05**
**J05**	**Antiviral drugs**	**7**	**1.1%**	**0.198 (0.094–0.419)**	**< 0.001**
**A04**	**Antiemetics and antinauseants**	**6**	**1.0%**	**1.922 (0.860–4.296)**	**> 0.05**

**C03**	**Diuretic drugs**	**6**	**1.0%**	**0.254 (0.113–0.570)**	**0.001**
**A09**	**Digestives, including enzymes**	**5**	**0.8%**	**0.127 (0.052–0.310)**	**< 0.001**
**A10**	**Drugs used in diabetes**	**5**	**0.8%**	**1.304 (0.541–3.146)**	**> 0.05**
**N01**	**Anesthetic drugs**	**5**	**0.8%**	**1.478 (0.612–3.565)**	**> 0.05**
**A05**	**Bile and liver therapy**	**3**	**0.5%**	**0.240 (0.077–0.750)**	**0.014**
**A11**	**Vitamins**	**3**	**0.5%**	**0.773 (0.248–2.070)**	**> 0.05**
**B03**	**Antianemic drugs**	**3**	**0.5%**	**0.233 (0.075–0.729)**	**0.012**
**C01**	**Cardiac therapy**	**3**	**0.5%**	**0.570 (0.183–1.775)**	**> 0.05**
**G03**	**Sex hormones and modulators of the genital system**	**3**	**0.5%**	**0.675 (0.217–2.102)**	**> 0.05**

Abbreviations: WHO-ATC: World Health Organization-Anatomical Therapeutic Chemical Classification System ; ROR: reporting odds ratio; CI: confidence interval; BLI: beta-lactamase inhibitor.

[a] WHO-ATC code shown up to the second level (bolded). [b] ROR from the Mantel-Haenszel test for fatal events compared to non-fatal events. [c] *p*-value from the Mantel-Haenszel test between fatal and non-fatal events.
